# A novel frameshift variant in the *ADA2* gene of a patient with a neurological phenotype: a case report

**DOI:** 10.1186/s12969-022-00781-9

**Published:** 2022-12-17

**Authors:** Z. Lucane, Z. Davidsone, I. Micule, M. Auzenbaha, N. Kurjane

**Affiliations:** 1 Riga Stradins University, Dzirciema Street 16, Riga, LV-1007 Latvia; 2 Children’s Clinical University Hospital, Vienibas Street 45, Riga, LV-1004 Latvia

**Keywords:** Deficiency of adenosine deaminase 2, Immunodeficiency, Stroke, Vasculitis, Autoinflammatory syndromes, Case report

## Abstract

**Background:**

Adenosine deaminase 2 (ADA2) deficiency is an inherited autoinflammatory syndrome caused by a defect in the *ADA2* gene. Most common manifestations include peripheral vasculopathy, early-onset stroke, immunodeficiency, and haematological manifestations. Patients with pathogenic variants that are more detrimental to ADA2’s enzymatic function (e.g. frameshift) have been reported to be prone to developing hematological phenotype. We report here the case of a 13-year-old Caucasian girl with a novel frameshift variant in the ADA2 gene and a clinical phenotype of early-onset stroke.

**Case presentation:**

The patient was admitted to hospital with complaints of weakness in her right arm, unilateral facial weakness and speech problems. Her initial laboratory workup was normal; however, magnetic resonance imaging of her brain confirmed acute/subacute ischaemic changes in the posterior limb of the left-sided internal capsule and in the apical part of the thalamus. She also had manifestations of immunodeficiency – recurrent skin infections and otitis, chronic *Molluscum contagiosum* infection in anamnesis and B cell deficiency with a low level of serum IgA. The patient’s DNA was analysed and two pathogenic variants were identified in the *ADA2* gene, confirming a diagnosis of adenosine deaminase 2 (ADA2) deficiency. While one of the variants (c.506G > A (p.Arg169Gln)) has been reported previously, the other one is a novel frameshift variant, namely, c.464del (p.Pro155Hisfs*29). The patient received stroke rehabilitation, which significantly improved her functional state. Tumour necrosis factor inhibitor and methotrexate treatment was commenced, and the patient has remained stable with no further ischaemic events.

**Conclusions:**

Although rare, ADA2 deficiency should be considered in patients with early-onset stroke, especially with concomitant manifestations of inflammatory features or immunodeficiency. This case report extends the genotypic spectrum of ADA2 deficiency.

## Background

Adenosine deaminase 2 (ADA2) deficiency is an inherited autoinflammatory syndrome that was first described in 2014 by two independent research groups as a monogenic vasculitis resembling polyarteritis nodosa in its clinical manifestations [1, 2]. The prevalence of the disease is estimated at approximately 1 in 222,000 [3]. To date, no cases of ADA2 deficiency have been reported in Latvia [4].

It is an autosomal recessive disease caused by pathogenic variants in the *ADA2* gene, which is located on chromosome 22q11.1. More than 100 disease-associated variants have been identified over the entire coding region [3]. Missense variants are the most common (such as p.Gly47Arg (p.G47R), p.Gly47Ala (p.G47A), p.Arg169Gln (p.R169Q), p.Tyr453Cys (p.Y453C)), but single amino acid deletions, insertions/deletions with frameshift, nonsense and splicing variants have also been described [3, 5].

The *ADA2* gene encodes the extracellular enzyme ADA2 which catalyses the conversion of adenosine and 2′-deoxyadenosine to inosine and 2′-deoxyinosine, respectively [6]. Deficiency of the enzyme results in increased M2 macrophage apoptosis, resulting in reduced M2 macrophage anti-inflammatory activity and increased M1 macrophage pro-inflammatory activity. In turn, this contributes to an inflammatory environment and vasculitis, most commonly manifesting as early-onset strokes and peripheral vasculopathy [7]. However, in recent years it has become evident that the spectrum of clinical manifestations is highly pleiotropic, with features such as autoinflammation, lymphoproliferation, immunodeficiency and haematological manifestations, which vary from milder manifestations including anaemia, neutropenia, and lymphopenia to transfusion-dependent pure red cell aplasia and bone marrow failure. In addition to multiple clinical manifestations, disease severity is also highly variable, even in members of the same family with identical gene defects. Thus, genotype-phenotype correlations are difficult to elucidate.

The use of tumour necrosis factor (TNF) inhibitors in the treatment of ADA2 deficiency has significantly improved patient care; however, the response to therapy is not always favourable, especially in cases with immunodeficiency and haematological manifestations. For patients with a severe haematological phenotype, allogeneic haematopoietic stem cell transplantation (HSCT) is available, and studies examining the role of autologous HSCT gene therapy have been published [8–10].

Here we describe the case of an ADA2 deficiency patient carrying a novel frameshift variant – c.464del (p.Pro155Hisfs*29) – in the *ADA2* gene and exhibiting a predominant phenotype of vasculopathy with no haematological manifestations.

## Case presentation

A previously healthy 13-year-old Caucasian girl was admitted to the Children’s Clinical University Hospital with complaints of weakness in her right arm, unilateral facial weakness and speech problems. Regarding the patient’s medical history, at the age of 2.5 years she was diagnosed with a patent *ductus arteriosus* which was corrected by transcatheter closure. Prior to the surgery, she had disseminated *Molluscum contagiosum* infection that was treated with electrocoagulation and left multiple scars. At pre-school age, she had motor tics (eye blinking). It was also documented that she had recurrent acute respiratory viral infections, skin infections of unknown aetiology, otitis and episodes of arthralgia of the elbow joints, usually lasting about 2 days. Her developmental history was compatible with her age and she had received all vaccinations according to the recommended schedule. The girl was born to non-consanguineous parents and her family’s medical history did not reveal any hereditary diseases. It was noted that one of her grandparents had a stroke at an elderly age.

On admission to hospital, the patient’s general physical examination was found to be normal: blood pressure 119/78 mmHg; pulse 70/min regular; respiratory rate 18/min; weight 56.2 kg (+ 0.5 SD); height 164.5 cm (+ 0.5 SD). She had multiple punctate scars on the arms and legs due to the contagious molluscum infection in personal medical history; no other skin abnormalities were observed. Neurological examination revealed elements of motor aphasia (she spoke in short phrases) and corticonuclear insufficiency on the right side: facial asymmetry to the right, more pronounced in cheeks and mouth area; otherwise, cranial innervation was intact - visual fields were normal in all quadrants, pupils were reactive to light and accommodation, eye movements unrestricted in all directions, tongue protruded midline and moved symmetrically. Pharyngeal reflex was positive. There was slight unilateral pronator drift of right arm on the right side. Muscle strength in the right arm was proximal 4 points, distal 3 points (MMT; Manual Muscle Testing). Active movement in the right shoulder were up to 100° flexion; there was limited forearm pronation/supination and extension of the fingers. Muscle strength in the right leg was 4 points, both proximal and distal. Muscle strength in the left arm and leg was normal (5 points). Coordination test was performed more inaccurately with the right hand, according to paresis. Tendon reflexes were symmetrical (2/4 bilaterally). Pathological reflexes were negative. Sensation was intact bilaterally. She was able to sustain balance with eyes closed (Romberg test). Meningeal symptoms were negative.

Her initial full blood count, biochemistry and coagulation testing did not reveal any pathology. A computed tomography scan of the patient’s head detected no abnormal findings. Cerebral magnetic resonance imaging was performed the next day and confirmed acute/subacute ischaemic changes in the posterior limb of the left-sided internal capsule and in the apical part of the thalamus. Magnetic resonance angiography showed no abnormal changes in the blood vessels and subacute ischaemia in the left posterior capsula interna and apical-lateral part of the thalamus, presumably in the branches of the left middle cerebral artery (lenticulostriate arteries). The patient was started on aspirin 100 mg a day.

Consequently, the possible aetiology of an early-onset stroke was investigated. To exclude a cardiac aetiology, electrocardiography was performed – no pathological findings were observed. Furthermore, transthoracic echocardiography with agitated saline did not detect a right to left shunt. Laboratory screening for hypercoagulable states was negative. Screening for infection was also negative (SARS-CoV-2, influenza, HIV-1/− 2 antibodies and HIV-1 antigen, viral hepatitis B and C, *Borrelia burgdorferi* IgM and IgG, *Treponema pallidum* IgM and IgG, TPHA, *Varicella zoster* DNA). No specific finding indicative of a primary metabolic defect was detected via determination of the spectrum of organic acids in urine. Rheumatological workup showed a slightly elevated anti-dsDNA, with other tested antibodies within the reference range. However, the rheumatologist suspected ADA2 deficiency and so the patient was referred to the genetics clinic.

Genetic testing was performed and sequence analysis using Blueprint Genetics’ Primary Immunodeficiency Panel (comprising of 336 genes) identified two variants in the *ADA2* gene: a missense variant c.506G > A (p.Arg169Gln) and a frameshift variant c.464del (p.Pro155Hisfs*29). The next-generation sequencing data indicated that these variants were on different parental alleles (in trans position) in the patient. The frameshift variant c.464del (p.Pro155Hisfs*29) is not recorded in the ClinVar [11] and HGMD [12] databases. This variant deletes one base pair in exon 3 (of a total of 10 exons) and generates a frameshift which leads to a premature stop codon at position 29 in the new reading frame.

After the ADA2 deficiency diagnosis was confirmed, the patient was consulted by an immunologist and subsequent laboratory immunological evaluation revealed decreased B cell absolute counts and low serum IgA with normal IgG and IgM (Table 1).

**Table 1:** Immunological testing

Laboratory test	Result	Reference range
CD3+ T cells, %	71.43	52–78
CD3+ T cells, 10^3^ μL	0.92	0.8–3.5
CD3+ CD8+ T cells, %	21.95	9–35
CD3+ CD8+ T cells, 10^3^ μL	0.28	0.2–1.2
CD3+ CD4+ T cells, %	48.56	25–48
CD3+ CD4+ T cells, 10^3^ μL	0.63	0.4–2.1
CD56/CD16+ NK cells, %	17.44	6–27
CD56/CD16+ NK cells, 10^3^ μL	0.22	0.07–1.2
CD19+ B cells, %	9.82	8–24
CD19+ B cells, 10^3^ μL	0.13	0.2–0.6
CD4/CD8	2.21	0.9–3.4
IgG, mg/dL	1152	716–1711
IgA, mg/dL	2	47–249
IgM, mg/dL	28	15–188

On discharge from the hospital, facial asymmetry had decreased, but elements of motor aphasia remained. Right-sided paresis remained, more pronounced in the hand. She was able to grasp an object with her right hand, but her fine motor and object manipulation skills in the hand were impaired. Stroke rehabilitation (physiotherapy, ergotherapy and audiologopaedic therapy) significantly improved the patient’s functional state – facial asymmetry was no longer observed and full motor function of the right arm was regained within 4 months. Neurological examination showed positive palmomental reflex on the right side, otherwise with no signs of focal neurological deficits. Following rehabilitation, she started the combined medication of adalimumab (TNF inhibitor) 40 mg twice a month and methotrexate 7.5 mg once a week (to reduce the risk of developing an antibody to adalimumab). At her 1.5-year follow-up, she continues to take the medication and has remained well with no further ischaemic events. Control MRI scan was performed and no silent brain infarcts were detected.

## Discussion and conclusions

The patient in this case report presented with an early-onset stroke and immunodeficiency. Genetic testing revealed two pathogenic variants in her *ADA2* gene, confirming a diagnosis of ADA2 deficiency. While one of the variants (c.506G > A (p.Arg169Gln)) has been reported previously [1], the other one is novel. Thus, we provide here the first description of a new frameshift variant in the *ADA2* gene, namely, c.464del (p.Pro155Hisfs*29).

The prevalent clinical phenotype of our patient was brain infarct in the internal capsule and thalamic region. Indeed, it has previously been reported that ADA2-deficient patients often have lacunar infarcts in the brainstem, thalamus, basal ganglia and internal capsule of the brain [6]. Our patient also had a B cell deficiency with normal T cell subsets, low serum level of IgA and clinical signs of immunodeficiency – disseminated *Molluscum contagiosum* infection, recurrent skin infections and otitis in early childhood. As reported previously, a compromised B cell compartment and hypogammaglobulinaemia are common features in ADA2 deficiency patients. Thus, differential diagnosis from common variable immunodeficiency (CVID) is important [13]. This is illustrated by Schepp et al.’s study of a cohort of 181 CVID patients in which nine patients with ADA2 deficiency were misdiagnosed as CVID patients [13]. Regarding immune dysregulation, our patient did not display any clinical signs of autoimmune disease, atopy or lymphoproliferation; however, she did exhibit a slight increase in anti-dsDNA autoantibodies. Arthralgia is also common, occurring in 31% of ADA2-deficient patients, and was present in our patient [6]. Dermatological involvement is observed in 75% of ADA2 deficiency cases, with the most common clinical presentations being *livedo racemosa, livedo reticularis* and Raynaud’s phenomenon [5]. However, in our patient severe disseminated *Molluscum contagiosum* infection was the main dermatological abnormality, and it was present at an early age prior to the stroke. Disseminated *Molluscum contagiosum* infection and recalcitrant warts have previously been described in a case series of two unrelated cases [14]. As reported previously, the two phenotypes of vasculitis and haematological disease tend not to overlap. This was also the case in our patient, who had no signs of cytopenia [15]. The clinical heterogeneity of ADA2 deficiency highlights the need to suspect this syndrome not only in early stroke but also in a variety of clinical conditions – unexplained rheumatological symptoms, unexplained cytopenia and immunodeficiency. Several factors have been proposed to contribute to this clinical variability, including residual ADA2 enzyme activity and variations in ADA2-binding receptors [15, 16]. In our patient, ADA2 enzyme catalytic activity was not assessed because the diagnosis was confirmed by genetic testing.

Although various pathogenic variants have been described in ADA2 deficiency, the genotype-phenotype correlations have been difficult to establish [16]. Nevertheless, patients with pathogenic variants that are more detrimental to ADA2’s enzymatic function (e.g. nonsense, frameshift) have been reported to be prone to developing severe cytopenia. In a study investigating genotype-phenotype correlations, more than 90% of pathogenic variants in the vasculitis group were missense mutations, compared to 53% of variants in the pure red cell aplasia group and 72% of variants in the bone marrow failure group. If compound heterozygous variants were excluded, all patients in the vasculitis group had missense variants, whereas more diverse gene variants were found in the haematological manifestation groups [15]. Interestingly, our patient had a frameshift variant and exhibited a predominant phenotype of vasculopathy with no haematological manifestations. Additional modifier genes, epigenetic variations and environmental factors can also contribute to differences in clinical manifestations [3, 16]. Furthermore, pathogenic variants in the dimerization domain of the *ADA2* gene have been found to associate with vascular manifestations, whereas pathogenic variants in the catalytic domain have been described to be associated with haematological manifestations [17]. Establishment of the genotype-phenotype correlations is also important for a patient’s therapeutic options. For instance, TNF inhibitor therapy has been reported to be more beneficial for patients with vascular inflammation in comparison to patients with haematological manifestations [10]. In line with this finding, a favourable therapeutic effect of TNF inhibitor therapy (adalimumab) was observed in our patient. Indeed, at her 1.5-year follow-up, she was in remission and continues to take the combined medication of adalimumab and methotrexate. Therefore, since viable therapeutic options are available for this inborn error of immunity, next-generation sequencing is pivotal for the early detection of this disease.

Although rare, ADA2 deficiency should be considered in patients with an early-onset stroke, especially with concomitant manifestations of inflammatory features or immunodeficiency.

## Data Availability

Data sharing is not applicable to this article as no datasets were generated or analysed during the current study.

## Abbreviations

ADA2: Adenosine deaminase 2
TNF: Tumour necrosis factor
HSCT: Haematopoietic stem cell transplantation
SARS-CoV-2: Severe acute respiratory syndrome coronavirus 2
HIV: Human immunodeficiency virus
TPHA: *Treponema pallidum* haemagglutination
anti-dsDNA: Anti-double stranded DNA
HGMD: Human genome database
NK: Natural killer cells
CVID: Common variable immunodeficiency
MMT: Manual Muscle Testing
